# The Role of Endocrine Disrupting Chemicals in Gestation and Pregnancy Outcomes

**DOI:** 10.3390/nu15214657

**Published:** 2023-11-03

**Authors:** Maria Puche-Juarez, Juan M. Toledano, Jorge Moreno-Fernandez, Yolanda Gálvez-Ontiveros, Ana Rivas, Javier Diaz-Castro, Julio J. Ochoa

**Affiliations:** 1 Department of Physiology, Faculty of Pharmacy, Campus Universitario de Cartuja, University of Granada, 18071 Granada, Spain; mpuchej@ugr.es (M.P.-J.); jjoh@ugr.es (J.J.O.); 2 Institute of Nutrition and Food Technology “José Mataix Verdú”, University of Granada, 18071 Granada, Spain; yolandagalvez@ugr.es; 3 Nutrition and Food Sciences Ph.D. Program, University of Granada, 18071 Granada, Spain; 4 Instituto de Investigación Biosanitaria (IBS), 18016 Granada, Spain; amrivas@ugr.es; 5 Department of Nutrition and Food Science, University of Granada, 18071 Granada, Spain

**Keywords:** endocrine disrupting chemical, pregnancy, gestation, complications, maternal-fetal health, fertility, bisphenols, phthalates, pesticides, advanced maternal age

## Abstract

Endocrine disrupting chemicals (EDCs) are exogenous substances widely disseminated both in the environment and in daily-life products which can interfere with the regulation and function of the endocrine system. These substances have gradually entered the food chain, being frequently found in human blood and urine samples. This becomes a particularly serious issue when they reach vulnerable populations such as pregnant women, whose hormones are more unstable and vulnerable to EDCs. The proper formation and activity of the placenta, and therefore embryonic development, may get seriously affected by the presence of these chemicals, augmenting the risk of several pregnancy complications, including intrauterine growth restriction, preterm birth, preeclampsia, and gestational diabetes mellitus, among others. Additionally, some of them also exert a detrimental impact on fertility, thus hindering the reproductive process from the beginning. In several cases, EDCs even induce cross-generational effects, inherited by future generations through epigenetic mechanisms. These are the reasons why a proper understanding of the reproductive and gestational alterations derived from these substances is needed, along with efforts to establish regulations and preventive measures in order to avoid exposition (especially during this particular stage of life).

## 1. Introduction

Several chemicals, both natural and synthetic, can interfere with different pathways of the endocrine system, altering its functioning and leading to detrimental consequences for human health. These compounds are known as endocrine-disrupting chemicals (EDCs), and can be found in a variety of products, including metals, pesticides, and daily products such as plastic food-packaging, flame retardants, toys, cosmetics, detergents, etc. [1]. They can interact with the endocrine system homeostasis in many ways, altering the synthesis, secretion, binding, transportation, metabolisms and/or elimination of key hormones. Their ubiquitous presence of these exogenous substances and their demonstrated or potential negative impact on health has turned them into an object of profound investigation by the scientific community, showing the risk that these compounds represent [2]. As a matter of fact, several institutions have been developing policies related to EDCs since the 1990s, with the objective of regulating, reducing, or even banning some of these chemicals’ applications. In accordance with the scientific research and findings, these policies have been increasingly hardened over the years, in order to reduce population exposure to them [3].

Among their wide effects, EDCs have demonstrated an ability to harm the reproductive system of both women and men. Their detrimental impact on reproduction starts even before the conception, affecting the fecundability of both parents [4]. However, their implications are more apparent during pregnancy since it is one of the most sensitive stages of life when it comes to environmental factors. EDCs may interact with the activity of many hormones involved in reproduction and development, which undoubtedly highlights the potential damage that EDCs can provoke to both mothers and their offspring [1]. These substances can reach pregnant women through different ways, including the food chain, the skin, or the respiratory tract, with several studies pointing out that EDCs can be detected in their urine, serum, breast milk and amniotic fluid. Additionally, some of them can accumulate in placental tissues, impairing their function [5]. The importance of this fact is related to the crucial role that the placenta exerts during pregnancy, as it assures fetal homeostasis by performing the exchange of nutrients, gasses, signaling molecules and waists needed for its development, as well as by acting as a protective barrier against external aggressions [3]. However, this transient organ is not completely impermeable, and some EDCs such as bisphenol A, phthalates and organochlorine pesticides are able to cross the barrier and reach the fetus. This developing organism is particularly sensitive to external agents, due to its immaturity and its huge cell differentiation rate, so little changes in hormone and protein levels would have a serious impact [3]. Consequently, dangerous complications and poor pregnancy outcomes may occur, including fetal growth restriction, low birth weight, preeclampsia, gestational diabetes mellitus (GDM), preterm birth and baby loss [6]. The prevalence of these complications has gradually augmented over the last decades, in which EDC exposure has also increased, highlighting a relationship between environmental exposure to these substances and this increasing prevalence [7].

However, the potential negative effects of these chemicals not only appear during the gestational process. Subtle endocrine, metabolic, and biochemical alterations related to EDCs may exert powerful and permanent effects on tissues in formation, changing the developmental trajectory of the fetus. These effects are referred to as “early programming” and represent an important susceptibility factor for the appearance of non-communicable diseases (NCDs) during postnatal life [8]. According to the “developmental origins of health and disease hypothesis” (DOHaD) described by Barker, preconception, pregnancy and the first two years of life have an impact on later-life health outcomes. In this sense, fetal development and delivery outcomes are predictors of health in adulthood [9]. In fact, apart from influencing different aspects of the gestation process, EDCs exposure has been previously associated with obesity, diabetes, and cardio metabolic risk, as well as with alterations in cognitive development and behavior [10,11], as some of them have proved to cross the blood-brain barrier of the immature fetus [12]. Moreover, it has even been proved that certain EDCs can create a predisposition in the exposed fetus for an eventual development of some types of cancer [13].

Even though several adverse effects have been documented, the actual damages on pregnancy, the placenta, and fetus health remain unclear. The scientific literature currently available provides, in some cases, contrasting results concerning the short- and long-term alterations that these chemicals would produce on the gestation process and its subsequent consequences, so further investigation needs to be carried out to clarify this subject.

## 2. Materials and Methods

Bibliographical research was performed, starting in February 2023, and continued until early April 2023, when the current review was carried out. The search was focused in the last 5 years (2019–2023), using the main biomedical databases and sources: Medline (via PubMed), The Cochrane Library, Elsevier, and Dialnet. Article acceptance has been only considered for relevant studies published in recent years, all of them related to the subject of the present study; alterations in fecundability, pregnancy outcomes, and complications associated with maternal exposure to EDCs. Higher attention was paid to those articles related to EDCs and pregnancy health. The search process was carried out just considering articles in English, as it is the scientific lingua franca. With regard to key words applied, these were: endocrine disrupting chemicals, bisphenols, phthalates, pesticides, polycyclic aromatic hydrocarbons, perfluorinated compounds, parabens, pregnancy, gestation, fertility, pregnancy complications, pregnancy outcomes. Medical subject heading (MSH) terms were used in those words that may create misunderstanding in the browser. Furthermore, the applied Boolean operators were “AND”, “OR” and “NOT”, having been combined with key words so as to find more relevant articles. “AND” was applied between every word to achieve a search with more sensitivity and specificity. “OR” was used to connect synonyms. “NOT” was not frequently used, in order to avoid confusion in the browser.

**Figure 1:**
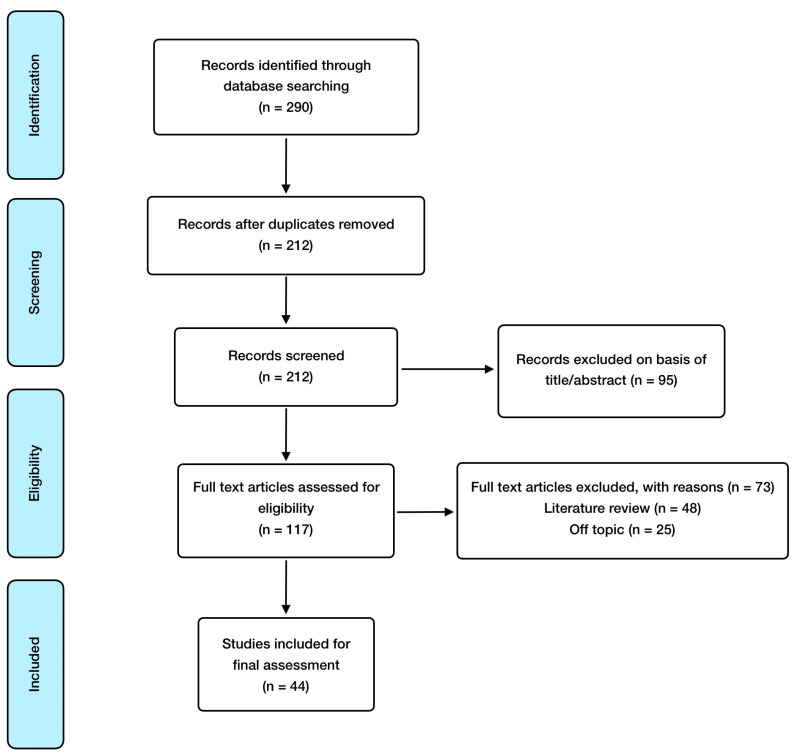
Manuscript selection flowchart.

As for inclusion criteria, these were the following: randomized controlled studies, observational studies, meta-analysis, animal model and in vitro studies; and English language, with special consideration for those articles involving pregnancy results derived from EDCs exposure. Exclusion criteria included the following: absence of abstract and publication in a language different from English. EndNote X8.2 was utilized as reference software for article management, citation, and bibliography organization. As for search methodology, the article selection process carried out is shown in Figure 1.

## 3. Results and Discussion

### 3.1. Endocrine Disruptive Chemicals (EDCs)

The World Health Organization (WHO) defines an endocrine disruptor as “an exogenous substance or mixture that alters function(s) of the endocrine system and consequently causes adverse health effects in an intact organism, its progeny, or (sub)populations” [4]. This heterogeneous group of substances (almost 800 chemicals) can alter the endocrine system mainly by mimicking, antagonizing, or modifying the metabolism of natural hormones, such as estrogens, androgens, and thyroid hormones [1]. Among the wide variety of EDCs, this review is focused on the compounds described below in this section, as they have been more deeply addressed with regard to their effects on fertility, pregnancy physiology, and fetal development [14]. The main types of EDCs regarding pregnancy development, as well as their abbreviations and exposure sources, are included in Table 1.

Bisphenols are industrial man-made chemicals widely utilized in the elaboration of polycarbonate plastics and epoxy resins. The first ones are usually used to fabricate bottles and food packaging, whereas the second ones are applied in composite materials to coat metal products, including cans or bottle tops. Consequently, diet would constitute the main source of exposure to these EDCs. However, other daily objects contain bisphenols in their composition, such as coating powders, medical material, or thermal printing papers, which translates in a wide contact with these chemicals through the environment [15]. They have low water solubility, being soluble in organic solvents, which makes them more easily accumulative within the lipid compartments of the body [3]. Bisphenol A (BPA) is the main and most used chemical of the group, being considered the first synthetic estrogen ever produced. The current tendency tries to substitute it with other supposedly less toxic analogs, such as bisphenol S (BPS), bisphenol F (BPF), bisphenol B (BPB), bisphenol AF (BPAF), tetramethyl bisphenol F (TMBPF), and tetrabromobisphenol A (TBBPA). However, their negative effects are similar to those exerted by BPA, having been demonstrated that BPS and BPF exhibit estrogenic and anti-androgenic activities which are equal and, in some cases, more powerful than BPA’s [16]. BPA is known for its estrogenic activity, which is exerted via activation of estrogenic receptors (ER), with a higher affinity for estrogen receptor β (ERβ). BPA monoglucuronide (BPA-G), its main metabolite produced in the organism, does not show this activity, but develops pro-inflammatory effects through the inhibition of peroxisome proliferator-activated receptor gamma (PPAR-γ) [17].

Phthalate esters (PAEs), also known as phthalates, are ubiquitous and synthetic diesters of phthalic acid with a useful activity as plasticizers, so they are applied to increase the flexibility, softness, and durability of several plastics, also being utilized as stabilizing agents. Food packaging, personal care products, toys, textiles, building materials and medical equipment are just some of the examples of the myriad products manufactured with these compounds [3]. They are some of the most widespread pollutants, having been found in almost 100% of tested subjects, and their levels seem to be higher in females compared to males [18]. These chemicals have demonstrated higher biological activity and toxicity after being metabolized to monoesters [19]. Diethyl phthalate (DEP) and di(2-ethylhexyl) phthalate (DEHP) are the most known molecules from this group, being monoethyl phthalate (MEP) the metabolite of DEP, while DEHP has a variety of them, such as mono(2-ethyl-5-hydroxyhexyl) phthalate (MEHHP), mono(2-ethylhexyl) phthalate (MEHP), mono(2-ethyl-5-oxohexyl) phthalate (MEOH), and mono(2-ethyl-5-carboxypentyl) phthalate (MECPP). As stated before, these metabolites activity has proved to be superior to that of their original compound [20]. On the other hand, there are also other phthalates and metabolites which have been less addressed by the scientific community, so they have yet to be further studied to assess their toxic effect in humans. These compounds include di(2-propylheptyl) phthalate (DPHP), di-iso-nonyl phthalate (DINP), benzyl butyl phthalate (BBP), di-n-butyl phthalate (DBP), dimethyl phthalate (DMP), di-n-octyl phthalate (DNOP), diisononyl cyclohexane dicarboxylate (DINCH), mono-(3-carboxypropyl) phthalate (MCPP), mono-iso-butyl phthalate (MIBP), monobutyl phthalate (MBP), mono-n-butylphthalate (MNBP), mono-benzyl phthalate (MBZP), mono-(2-ethyl-5-oxohexyl) phthalate (MEOHP), monocarboxy-isononly phthalate (MCNP), and mono-carboxy-isooctyl phthalate (MCOP) [4,21].

Other important EDCs to take into consideration are organochlorine pesticides (OCPs), which are organic chemicals containing carbon, hydrogen, and chlorine atoms in their composition. They have been used as pesticides for the control of insects, fungus, and weeds, representing 40% of all pesticides applied worldwide. This group of compounds include the widely banned (although not in every country) dichlorodiphenyltrichloroethane (DDT) and its metabolites; hexachlorobenzene, dieldrin and lindane [22]. They have poor water solubility and are highly soluble in organic solvents, being able to accumulate in the adipose tissue as many other EDCs. These molecules also have stable chemical properties, and they can adsorb organic compounds in soil, leading to its degeneration and contributing with water and air pollution. Another negative feature of these persistent organic pollutants (POPs) is their ability to be transmitted throughout the food chain, mainly from fish, and eventually, to humans [22]. Moreover, many of them have proved to be capable of crossing the placenta and reach fetal circulation, with the high risk that this can involve [23]. Another type of pesticides are organophosphates (OPs), which are esters of phosphoric acid utilized as insecticides due to their interference with acetylcholine neurotransmission. These are not as persistent in the human body, and the scientific evidence on them regarding pregnancy effects is limited [24]. Something similar happens regarding pyrethroids, which are being increasingly used as substitutes of other presumably more toxic pesticides [25].

Within the group of polycyclic aromatic hydrocarbons (PAHs), polychlorinated biphenyls (PCBs) are synthetic chemicals used in the fabrication of building materials and electrical equipment. Even though they have also been banned in several countries, contact with previously manufactured products containing them is still a source of exposure, as they are often used in chemical mixtures including a number of PCBs. The estimated half-life of these EDCs is 10–15 years and they show an accumulative environmental persistence, resulting in long-term effects in humans [26]. With a similar chemical structure, polybrominated diphenyl ethers (PBDEs) are also persistent PAHs utilized as flame retardants in a lot of products, including plastics, paints, textiles, furniture, foams, and electrical equipment. More than 200 of these compounds are currently known, and among them, less brominated chemicals are prone to accumulate in animals owing to their high lipid affinity, thus exerting a major toxic potential [27].

Perfluorinated compounds (PFCs), also known as perfluorinated alkylated substances (PFASs), undoubtedly have a relevant effect in endocrine disruption and have been frequently used in the production of non-stick cookware, firefighting foams, waterproof clothing, and anti-fouling paints. Perfluorooctane sulfonate (PFOS), perfluorooctanoic acid (PFOA and perfluononanoic acid (PFNA) are the most studied members of this group of chemicals, even though others may be more persistent in the environment [28]. Nevertheless, they are persistent as well, with half-lives of about 3.4 and 2.7 years respectively, which makes exposure before and during pregnancy of particular concern [29]. Parabens are p-hydroxybenzoic acid (PHB) esters, which have a preservative use in personal care products owing to their antimicrobial properties [30], also exerting endocrine disruptive effects, particularly in children [31]. Finally, phytoestrogens are plant components with weak estrogenic activity due to their low affinity for the estrogenic receptor, and are present in several plants, including beans, grains, fruits, vegetables, and particularly, soy and their derivative products. The three main types of phytoestrogens are isoflavones such as genistein, daidzein and glycitein; coumestans such as coumestrol; and lignan [32]. Table 2 summarizes the main information concerning those articles finally included and reviewed, referring to the addressed EDC, the study design, and the major findings derived from it.

### 3.2. EDCs and Fertility

Pregnancy success is undoubtedly linked to both male and female reproductive health. Unfortunately, exposure to EDCs, especially BPA and PAEs, has been reported to affect both genders’ fertility, compromising the viability of a possible pregnancy. With regard to women fertility, in vivo studies carried out in mice and rats have demonstrated that prenatal exposure to BPA inhibits germ cell nest breakdown, reduces the number of healthy follicles and decreases estradiol levels in F1 generation [77,78]. It has also been observed that this molecule can start a premature activation of primordial ovarian follicles [79]. On the other hand, BPA urinary concentration has been assessed in a prospective cohort study in order to find a relationship with ovarian response in women who are undergoing in vitro fertilization. The study reported an inverse association between BPA urinary levels and the number of oocytes retrieved per cycle [80]. Additionally, Wang and colleagues [81] concluded a 30% reduction in fecundability among women with the highest BPA urinary levels (from 700 subjects), together with a 64% increase in infertility odds.

PAEs have also demonstrated a negative effect on female fertility when it comes to oocyte maturation and development. In animal models, exposure to DEHP led to a reduction in the percentage of primordial follicles and an increase in the number of primary follicles. Therefore, the reproductive lifespan of these mice was significantly decreased [82]. This prenatal exposure to DEHP has also been associated with DNA methylation defects in several genes related to oocyte development, including insulin-like growth factor 2 receptor (IGF2R). These epigenetic alterations were discovered to be heritable, as they also appeared in the F1 and F2 generation of these mice [83]. However, Lopez-Rodriguez and colleagues [33] reached a slightly different outcome, as their study concluded that delayed pubertal onset and altered folliculogenesis was inherited by F2 and F3 mice, but not by F1 ones, after gestation exposition to a combination of EDCs, including BPA and phthalates (DEHP and DNBP), among others. These effects were identified as a result of an epigenetic alteration of hypothalamic genes controlling puberty and ovulation. In addition, their research showed a reduction in maternal behavior in F1–F3 mice, which was induced by a loss of hypothalamic dopaminergic signaling.

As for male fertility, EDCs have been widely related to negative affectation of men’s reproductive health, including modifications in testicle morphology and endocrine function, increase in DNA damage in sperm, and dampened semen quality [17,84]. This is supported by recent research in which BPA-treated mice reported decreased sperm quality, as well as lower serum testosterone levels, which meant a sub-fertile phenotype characterized by reduced fertilization efficiency and low pregnancy rate [34]. On the other hand, azoospermia in men with idiopathic infertility has been associated with noticeably high levels of plasma BPA (>3 ng/mL) [35]. A significant positive relationship between BPA levels in urine and LH concentration has also been found, together with an inverse association with sperm concentration in young college students. This means that this endocrine disruptor would be related to decreased Leydig cell capacity and sperm count, drastically reducing their fertility [85]. BPA has also been associated in a similar way with decreased sperm mobility, augmented percentage of immature sperm, and reduced antioxidant levels in this sample [17,85].

Phthalates have also reported an adverse impact on this matter. DEHP exposure in mice can induce lower testosterone serum levels, as well as higher concentrations of estradiol and LH. These mice also presented increased germ cell apoptosis, oligozoospermia and degeneration of seminiferous tubules. All these findings, together with histological evaluations, highlight a premature senescence in male reproductive health [86]. Alterations caused by PAEs seem to be inheritable, since Yuan and colleges found out that DBP exposure reduced sperm count in F1 to F3 generations, which was related to an epigenetic change in an important modulator of spermatogenesis and Sertoli cells activity [87]. Transgenerational effects after embryonic exposure to phthalates seem to be present up to F4 offspring generation, with alterations in spermatogonia, stem cell and testicular germ cell organization and functioning, together with reduced sperm count and motility, in this case, after DEHP administration [88].

As a conclusion, BPA, PAEs, and BP could have serious effects on the reproduction process, impairing both male and female fertility, and even causing gamete alterations that might be inherited by the offspring and their future descendants.

### 3.3. EDCs and Pregnancy

#### 3.3.1. Implications of Bisphenols in Pregnancy

The relevance of this EDC in the gestation process has been widely assessed. BPA can be related to a limitation of blood supply to both placenta and fetus, as some mice model studies have shown that exposure during early pregnancy may result in insufficient remodeling of uterine spiral arteries, with negative consequences for flow and vasoconstrictive ability [89]. This fact has been linked not only to lower placenta weight, but also to fetal intrauterine growth restriction-like [36] and preeclampsia-like phenotypes [37], which will be discussed in the following paragraphs.

The analogue BPF has been significantly associated with high probability of spontaneous abortions in rats, when administered at high doses [38]. There is scarce data on BPS (the main substitute of BPA in its current applications) effects, though placental dysfunction has been reported in sheep, with a decrease in binucleate cells (the equivalent of human syncytiotrophoblasts) [39]. The study of BPS toxicokinetic has revealed an elevated half-life in fetal circulation, even though its exposure is lower than BPA’s [90]. Tetramethyl bisphenol F (TMBPF), another analogue and potential substitute of these compounds, has proved to be even more toxic and teratogenic for chick embryos than BPA, which is the molecule intended to substitute [40].

BPA exposure during early fetus development can be associated with several abnormalities. For instance, BeWo cell in vitro studies have reported a rise in the expression of related syncytia proteins after exposure, which impairs trophoblasts fusion and their endocrine activity [41]. Human placental cell exposure to BPA has been reported to damage cell membranes, which can lead to negative outcomes in the gestation process, including preeclampsia, premature birth or even pregnancy loss [91]. BPA induces an altered trophoblast development, which in turn affects human chorionic gonadotropin (hCG) secretion by these cells and increases their apoptosis [92]. Nevertheless, other similar studies point out the opposite effect on apoptosis by increasing the production of some anti-apoptotic factors [93]. This has also been reported in trophoblast cell lines and explains that very low concentrations of this EDC reduce extravillous trophoblast invasion and migration through an alteration in hCG secretion [94]. The interference with hCG may be of great relevance, as it is required for maintenance of the corpus luteum, progesterone production, trophoblast differentiation and invasion, placental growth, and uterine angiogenesis [92]. hCG is not the only hormone whose secretion gets altered by bisphenols, since low doses can activate ERK signaling pathway, leading to reduction in placental aromatase activity, and consequently, a depletion in estradiol and progesterone synthesis. This hormone lack might possibly contribute to placental insufficiency and the subsequent pregnancy failure [95]. On the other hand, a recent study found no relationship between BPA levels and β-hCG production, but these results were attributed to the low BPA concentrations found in subjects, in comparison to those measured in other studies [42].

Some of these modifications associated with BPA are frequent in severe pregnancy syndromes such as preeclampsia. Even though the etiopathogenesis of this complication remains unclear, it seems to be produced by an imbalance between pro- and anti-angiogenic factors, including placental growth factor (PGF) and soluble fms-tyrosine kinase-1 (sFlt-1). According to this, maternal urine BPA levels have been related to increased sFlt-1/PGF ratios, specifically by increasing sFlt-1 levels [96]. This has been supported by mouse models in which exposure to BPA lowest effective dose led to hypertension, altered sFlt-1/PGF ratio, circulation abnormalities, and kidney failure, being all of them preeclampsia typical features. Decreased trophoblast invasion was also found, as well as increased metalloproteinases inhibitors and decreased metalloproteinases, which are key factors for cell fate regulation [37].

As for pregnancy duration, some authors have shown that BPA exposure leads to a higher likelihood of preterm deliveries [97], though some others disagree [98]. A study carried out by Huang et al. [43] reported that higher average concentrations of BPA across pregnancy were related to a 1.97-day decrease in the gestation process. Likewise, BPA levels in three trimesters were also negatively correlated with gestational age and positively associated with preterm birth in this study. In addition, a recent meta-analysis also concluded that there is a significant association between preterm birth and high BPA exposure levels [44]. Something similar happens regarding low birth weight and BPA. Some studies have found a negative relationship between these two variables, not only when BPA is determined in maternal blood, but also in amniotic fluid, highlighting placental permeability to this EDC [98,99]. Nevertheless, other researchers found that this correlation was possibly not clinically relevant enough [97], or even that there was no significant association [99]. On the other hand, Mustieles et al. [45] reported a strong relationship between preconception BPA levels and newborn size, which was believed to be mediated by germ cells-derived causes, possibly through epigenetic changes. Additionally, a prospective cohort study carried out on 788 mothers showed that BPA exposure was negatively related to intrauterine growth [100], while previous research found no association [101]. Supporting this, a recently published study suggests an inverse relationship between bisphenol mixtures and birth weight [46].

A link has also been established between BPA and GDM. EDCs exposure has been related to weight gain during pregnancy, and excessive weight gain in pregnant women is a renowned risk factor for the development of GDM. Specifically for BPA, results are not clear enough, since latest research highlights a positive association or lack of significant relevance when it comes to linking these two variables, rather than a negative impact. In a prospective cohort study, BPA urine levels correlated with lower pregnancy weight gain [47]. Likewise, Wang and colleagues reported that plasma glucose at 2 h in the 75-g oral glucose tolerance test was lower for women with higher concentrations of BPA. On the other hand, no statistically significant association between BPA exposure and impaired glucose tolerance or GDM diagnosis was found in other similar cohort studies [102]. Results from recent studies are rather contradictory, as they found out that the joint effect of bisphenols was positively correlated with the susceptibility to GDM. However, while BPS showed positive association, BPA’s and TBBPA’s reported to be negative [48].

**Figure 2:**
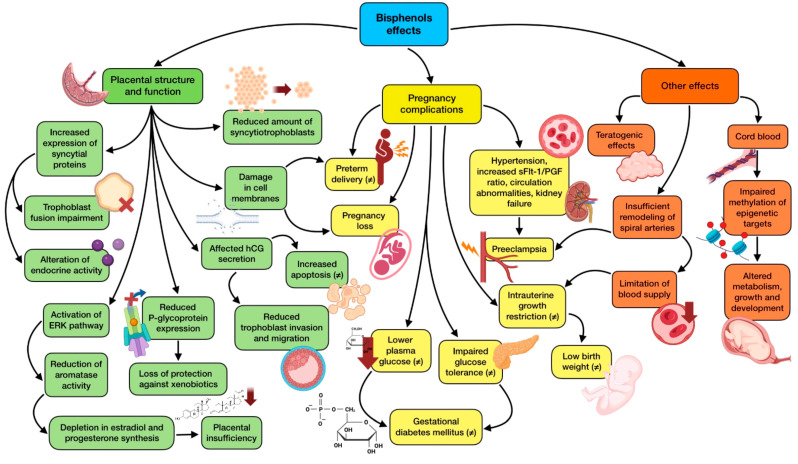
Summary of bisphenols effects on gestational development and complications. The existence of greater controversy on the effect due to contradictory results is indicated with (≠).

Many of the mentioned changes induced by BPA in the placenta are the result of a modification in DNA methylation and gene expression, which is translated in a negative affectation of placenta functioning and fetal health through epigenetic changes. Therefore, DNA methylation in newborns is predicted by BPA exposure during gestation, influencing the susceptibility of disease in the newborn [103]. Early fetal development is especially vulnerable to epigenetic alterations, due to the high DNA synthesis and the establishment of the complex system of DNA methylation, acetylation, and chromatin organization that is taking place. This fact highlights the importance of some epidemiological findings, which demonstrate an association among total placental BPA levels and global methylation in this organ [104]. Given the difficulty of getting samples from the first trimester of pregnancy, research on EDCs and placental-fetal epigenetics is focused on animal and in vitro studies. They show a negative relationship between maternal BPA and phthalate exposure in the first trimester and cord blood methylation of epigenetic targets tightly related to development, growth, and metabolism. Interestingly, a sex-stratified analysis revealed that these alterations were female-specific, which means that EDCs have sexually dimorphic effects regarding epigenetics [105]. Furthermore, data collected from human fetus liver biopsies showed that BPA exposure, even low doses, relevantly influenced epigenetic regulation of xenobiotic metabolizing enzymes, such as COMT and SULT2A1, which could lead to illness susceptibility later in life [106]. BPS has also been reported to have an epigenetic role, eventually reducing placental P-glycoprotein expression, one of the main efflux transporters that protects the fetus from xenobiotics present in maternal circulation. These effects seemed to be dependent on the type of exposure, with differences between acute and chronic [107]. Figure 2 summarizes the main alterations that bisphenols produce in the development of the gestation process, together with the appearance of possible pregnancy complications.

#### 3.3.2. Implication of Phthalates in Pregnancy

Phthalates can cause indirect placental alterations mediated by thyroid hormones, which are key regulators of this organ development. PAEs can downregulate the expression of Thrα1 and Thrβ1 mice genes, whose encoded proteins are thyroid hormone receptors (THRs), thus impairing the regular growth and functioning of the placenta and inducing fetal growth restriction. Moreover, these mice also presented a downregulation of THR downstream genes fundamental for placental angiogenesis, including vascular endothelial growth factor (VEGF), insulin-like growth factors 1 and 2 (IGF1, IGF2) and PGF [108]. These effects on the thyroid system are supported by recent human studies on this subject, in which higher DEHP levels were related to lower free thyroxine (FT4), while di-iso-nonyl phthalate (DINP) was associated with decreased total thyroxine (TT4), as well as low TT4/FT4 and total thyroxine/triiodothyronine (TT4/TT3) ratios. Other phthalates which are currently being introduced to replace traditional ones, were also associated with lower thyroxine and triiodothyronine (T4/T3) ratio, and higher FT4/TT4 and FT3/TT3 ratios [49].

Histopathological observations have revealed a decrease in the placental labyrinth layer (an analogous to human syncytium) of PAEs exposed rodents, which is related to pregnancy complications such as intrauterine growth restriction and preeclampsia [21]. It has been demonstrated that after DEHP exposure, placental gene expression gets altered, modifying apoptosis, fatty acid homeostasis, and angiogenesis, which leads to irregularities in vessel formation. Nevertheless, doses applied exceeded the population daily exposure and the tolerable daily intake for this phthalate [108]. Another potential alteration derived from phthalate exposure is lipid imbalance, as accumulation of glycerolipids and glycerophospholipids and impaired lipid metabolism in rat trophoblasts has been observed, hypothesizing that these compounds may inhibit estrogen and progesterone receptor binding [109]. Regarding placenta morphology, it gets relevantly affected by phthalates. A study carried out by Zhu and colleagues [110] concluded that prenatal exposure was related to altered size and shape, making it thicker and rounded. This relationship seemed to be more apparent in male newborn cases, supporting the hypothesis that placenta is a strong contributor to illnesses sexual dimorphism. Urinary DEHP has been inversely correlated with placental weight, which eventually leads to its insufficiency and intrauterine growth restriction [45,50], while MEHP exposure has been related to impaired hCG production [51] and increased corticotropin releasing hormone (CRH) expression [111].

PPAR-γ activation is one of the mechanisms phthalates exert in the placenta, through which MEHP has demonstrated to inhibit trophoblast invasion, thus augmenting the probability of pregnancy loss [112]. Previous studies support these findings, having reported an association between exposure to high doses of MEHP and early miscarriage [113]. Likewise, DEHP exposure is also believed to raise the susceptibility of early embryo loss [114]. In a recent study carried out in 463 women, higher quartiles of DEHP were strongly related to greater risk of recurrent pregnancy loss, mainly due to their antiandrogenic effect and their ability to bind ERα, showing detrimental effects on the female reproductive system [52]. As for preterm births, conclusions derived from studies assessing PAEs exposure are rather inconsistent, with authors reporting decreased [115], increased [116] or not affected [117] gestational age at delivery. The results obtained from different studies on the influence of PAEs exposure in low birth weight are also quite heterogeneous, up to the point that some of them indicate a positive relationship [118] while others failed to find this association [116,119]. Quite recently, a case-control study found out that DEHP, BBP and DMP were positively correlated with fetal growth restriction risk, while the opposite relationship was found for MBP, even though this last association was only demonstrated among female newborns [53]. Additionally, increases in urinary levels of phthalate metabolites have been observed to show an association with higher odds of preterm birth, ranging from 12% to 16% [54], which is supported by another prospective cohort study of sub-fertile couples in which maternal higher exposure to DEHP metabolites augmented the risk of preterm delivery [55].

With regard to preeclampsia, a recent prospective cohort study involving 1233 women showed a relationship among augmented urine phthalate concentrations and higher sFlt-1/PGF ratio. Nevertheless, no associations were found on other parameters, such as placental hemodynamic outcomes, maternal blood pressure, or gestational hypertensive alterations [56]. Other studies reported that increased levels of MEP, MCPP, and MIBP were associated with higher diagnosis of pregnancy-induced hypertension. Likewise, DEHP, MBP, and MEP were also correlated with augmented systolic blood pressure across the gestation process [57]. A recent meta-analysis addressed several PAEs in order to find an association with gestational hypertension, being MEP the only one that showed a positive relationship in this regard [58]. As for GDM, a large pregnancy cohort measured first trimester urinary phthalate levels (MEP and DEHP), associating them to gestational weight gain (which, as stated in the BPA section, is related to the appearance of GDM). This study also found a relationship between MEP exposure and increased susceptibility to impaired glucose tolerance, even though the association for DEHP was inverse [120]. Some studies did not find any link between urinary phthalates and IFG nor GDM [102], while others found a relationship depending on the type of phthalate: MEP was associated with GDM, and MNBP with IFG, though MCPP was inversely associated with GDM [59]. Additionally, MIBP and MBZP higher levels have been related to lower blood glucose concentration at the time of GDM diagnosis compared with lower levels, which would be a positive effect [121]. Research continues to be contradictory on this subject. A recent meta-analysis highlights a positive association between PAEs exposure and GDM [60]. In addition, a Chinese cross-sectional study reported that women with GDM showed higher MEHP urine levels. Interestingly, the MEHP dose-response relationship was not the same between mothers < 35 years of age and those aged > 35 years old [61]. On the contrary, a recent study failed to find an association between phthalates and maternal glucose outcomes, although it reported a correlation between MEP concentrations and higher odds of gestational weight gain [62].

Phthalates, and especially MEHP, can induce reactive oxygen species (ROS) generation and alter the expression and functioning of antioxidant enzymes, which in turn results in potentially serious DNA damage [122]. These changes seem to be mediated by PPAR-γ activation and subsequent link to retinoid X receptor (RXR) and migration to the nucleus [109]. It must be mentioned that oxidative stress evoked by some ROS such as H_2_O_2_ can modify mRNA and miRNAs expression, which is crucial for placental development. Oxidative DNA damage would eventually impair the binding of methylation proteins, causing relevant epigenetic changes in chromatin [123].

**Figure 3:**
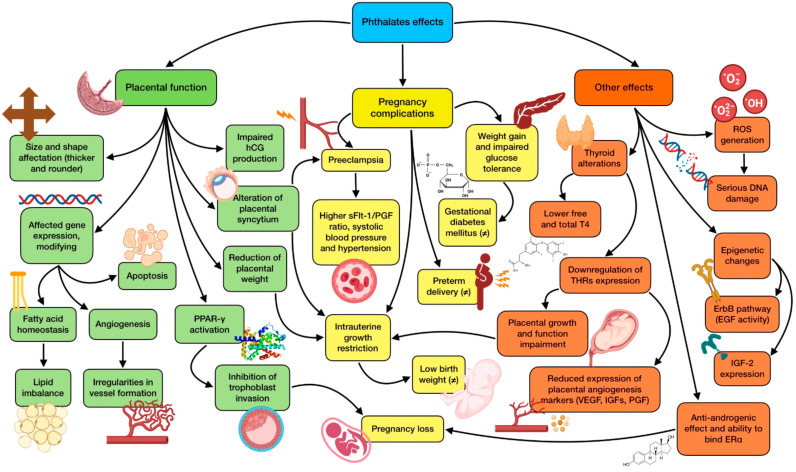
Summary of the main effects of PAEs on gestational development and complications. The existence of greater controversy on the effect due to contradictory results is indicated with (≠).

In this sense, the influence of PAEs on epigenetics has been studied to some extent. Exposure has been associated with methylation changes of 39 genes within the placenta during the first trimester, being these changes negative in most of the cases [124]. ErbB is one of the most relevant signaling pathways affected after DNA methylation induced by these EDCs. It involves the activity of epidermal growth factor (EGF) and epidermal growth factor receptor (EGFR), which are deeply associated with trophoblast proliferation, differentiation, and invasion; and consequently, placental growth and functioning [3]. Due to their abundance in placental tissue and their crucial role in early development, the consequences of these epigenetic changes should be a matter of deeper research. Placental transcriptome seems to be noticeably affected by phthalate exposure, inducing important epigenetic changes, affecting for instance, IGF2 expression (a major regulator of fetal and placental development) [124]. In this regard, urinary MEOHP and MEHHP levels have reported a negative correlation with IGF2 DNA methylation, especially when fetal growth restriction takes place [125]. Phthalate metabolites also seem to modify the methylation of Maternally Expressed 3 (MEG3) gene, which is tightly linked to early growth, metabolism, and tumorigenesis, according to a longitudinal birth cohort study carried out by Tindula et al. [126]. The most relevant effects of phthalates on pregnancy and possible associated complications are shown in Figure 3.

#### 3.3.3. Implications of Pesticides in Pregnancy

Organochlorine pesticides (OCPs) have been previously studied to assess their influence on pregnancy. The lipophilic nature of these compounds makes them easily dissolved and accumulated in human adipose tissue, being mobilized during pregnancy, reaching maternal blood and eventually, the placenta [127]. These EDCs can modify cytochrome P450 1A1 (CYP4501A1), which is involved in OCPs own metabolism. CYP4501A1 is also related to placental metabolism, so its altered expression might disrupt the detoxification ability of this organ. In addition, OCPs metabolism releases a big amount of ROS, which in turn affects DNA through a strand breakage and impairs mitochondrial functioning [128,129]. This excess of oxidative stress has demonstrated to increase the probability of preterm deliveries [63], as studies measuring γ-HCH (lindane) and β-HCH (lindane subproduct) in serum and cord blood reported that higher levels of these molecules were positively correlated with miscarriage and premature birth respectively [63,130]. OCPs can modify placental functioning, impairing hormone secretion and nutrient transport, which in turn disrupts fetus development [3]. In fact, studies have found that OCPs placental concentrations were negatively associated with several parameters of newborn development, including head and chest circumference, and body mass index [131]. They have also been reported to disrupt neonatal thyroid hormone status, with a detrimental impact on the subsequent growth process [132]. In this sense, quite recent research has reported lower birth weight in geographic areas where EDC profiles were dominated by relatively high OCPs levels [64].

As for DDT, even though its use has been long prohibited in several countries, its nature as a persistent organic pollutant makes it a widely spread substance in the environment. When assessing the placental effects of DDT, in vitro studies showed that high exposure leads to reduced cell viability, even though low doses do not modify cell proliferation. Modifications in enzyme expression were also reported, together with an impairment in the secretion of several hormones, such as estradiol, progesterone, and oxytocin [133]. Inhibition of aromatase activity has also been observed in previous studies, which significantly affects estrogen synthesis [134]. DDT is also involved in epigenetic alterations of placental genes, especially on IGF2, which might be related with fetal growth restriction complications [135]. On the other hand, DDT exposure has been previously related to maternal hypertensive alterations [136]. When it comes to birth weight, results are rather conflicting, reporting positive, negative, or even no correlation [137,138,139]. Nevertheless, elevated p,p′-DDE (DDT subproduct) has been measured in the blood of mothers exposed to this EDC, with subsequent transfer to umbilical cord blood and breast milk. Among its toxic effects on placenta and breast functioning, endothelial degeneration and vascular lesions must be highlighted [26].

With regard to organophosphates (OPs), evaluation of their placental effects is mainly restricted to chlorpyrifos and methyl parathion. Gestational exposure to the second one has reported a decrease in trophoblast giant cells and an augmentation in phagosome vacuoles within the labyrinth layer in rats. However, doses over reference doses (RfD) for human exposure were applied for these EDCs [21]. As for chlorpyrifos, even micromolar exposure has proved to be cytotoxic for placental cells, being able to trigger apoptosis by modulating tumor necrosis factor (TNF). It also impairs the expression of ABCG2 transporter (a pregnancy maintenance marker) and β subunit of hCG, although it does not seem to affect estradiol or progesterone production [140]. Furthermore, oxidative stress gets affected by chlorpyrifos exposure, with an increased ROS production and consequently and upregulation of endoplasmic reticulum proteins related to oxidative stress [141].

Pyrethroid pesticides, which are being used as substitutes of other more toxic molecules, are not lacking in pregnancy-related side effects. For instance, fenvalerate has shown to induce fetal growth restriction through an impairment in THRs pathways in mice [25].

#### 3.3.4. Implications of Polycyclic Aromatic Hydrocarbons (PAHs) in Pregnancy

Among these chemicals, PBDEs are to be highlighted. They can be found in several samples related to pregnancy, including placental tissue, cord blood, and breast milk, where they accumulate and are eventually transferred to the developing fetus [142]. Sex seems to be relevant for this EDC, as measured levels have reported to be significantly higher in the placentas of male newborns [143]. These molecules have proved to be toxic for cytotrophoblasts, leading to impaired viability, reduced migration, and invasion, altered lipid metabolism and apoptosis [65]. One of the most studied PBDEs regarding placental function is BDE-47, having also reported notorious effects on oxidative stress and increased CRH levels, which has been related to premature delivery [144].

Results on fetal weight are rather discordant when it comes to PBDEs exposure. Many epidemiological studies report a negative association, some of them, non-significant enough, only found on male infants, whereas others reported a lack of statistically significant association [145]. Nevertheless, a meta-analysis carried out by Zhao and colleagues [146] found a relevant negative correlation regarding PBDEs cord blood concentration and birth weight. Exposure to PBDEs during pregnancy has also reported a relationship with alteration of normal placental growth, measuring these compounds in cord blood [147]. In addition, they have shown to modify miRNA expression, leading to preeclampsia-related changes [148,149]. Recent research on preterm births showed an association between high concentration of PBDEs and shorter gestational age at delivery and spontaneous preterm birth [66]. On the other hand, a relevant health component that has shown to be disrupted by EDCs during pregnancy is the inflammasome. During the first trimester, IL-6 elevation has been described in relation to environmental exposure to different EDCs mixtures, including phthalates and PBDEs [67]. This IL-6 increase has previously been associated with dampened IGF bioavailability within the placenta, and in turn, with fetal growth restriction [150]. In fact, PBDEs have previously been related to impaired IGF-1 content in the placenta [151].

As for PCBs, exposure to doses over the RfD can disrupt the labyrinth layer in rats’ placentas [152]. Nevertheless, and despite PCBs hormone-related effects, human studies have failed to find a significantly strong association between these EDCs and pregnancy negative outcomes such as spontaneous abortion [153]. High placental PCBs levels have been related to lower syncytiotrophoblast volume and increased PGF expression in humans, inducing vessel formation and spiral artery remodeling [154]. However, similar research studying PCBs mixtures has reported anti-angiogenic effects in the maternal-fetal interface, together with induction of placental cell apoptosis [155]. On the other hand, PCBs concentrations have been inversely correlated with birth weight [156], and a recent meta-analysis has reported a significant influence of PCBs on the incidence of GDM, which was also found for PBDEs as well [60].

#### 3.3.5. Implications of Perfluorinated Compounds (PFCs) in Pregnancy

PFCs have been evaluated in animal model studies to investigate their effects on pregnancy. They showed that exposure during mid- to-late gestation results in lower placental and fetal weight. Also, an increased probability of placental necrosis was reported, together with an increase in fetal corticosterone levels and the inhibition of steroid related enzymes. Nevertheless, doses applied were higher than established RfDs for humans [157,158]. Research in human cytotrophoblast cell lines highlights that these compounds seem to modulate steroid hormone signaling, through a reduction of aromatase production, estradiol liberation and progesterone generation. As for PFOS, exposure to this chemical leads to lower cell viability and apoptosis [159]. Other studies have reached the conclusion that developmental exposure to PFOA negatively affects fetal growth and birth weight [160]. More recently, this negative effect has been deeper established for both PFOS and PFOA [161].

Several cohort and case-control studies have highlighted a concern about the contribution of PFASs exposure to the development of GDM and impaired glucose tolerance [68,69,162,163,164]. A meta-analysis has also supported this positive relationship between PFASs and GDM risk [60]. Additionally, PFCs concentrations in umbilical cord blood have been positively correlated with preeclampsia events [70]. This has been supported by another meta-analyses, which reached the conclusion that PFOA, PFOS and PFNA were significantly associated with augmented risk of preeclampsia, together with a significant relationship between PFOA and gestational hypertension [58]). Finally, epidemiological studies have proved that PFCs can enter fetal blood circulation, which seems to take place prenatally, through placental transport, and postnatally in the newborn, through breastfeeding [165].

#### 3.3.6. Implications of Phytoestrogens in Pregnancy

Phytoestrogens exert a weak estrogen-like activity through the binding to ERs located in many tissues, including reproductive tract, placenta, and mammary glands [166]. The effects of the different types of ERs are distinct too, since ERα usually promotes cell growth while ERβ frequently induce apoptosis; and phytoestrogens tend to present different affinities for them. For example, coumestrol, genistein, and apigenin have higher affinity for Erβ [3]. Exposure to these compounds during sensitive stages of development may be able to modify the reproductive system functioning, thus affecting pregnancies and fetal health. As for genistein, this molecule has reported the capacity to alter PGF and IGF-1 levels, thus impairing the regular development of both the placenta and the fetus [71]. This phytoestrogen, together with daidzein, can also inhibit the secretion of hCG by trophoblast cells [167].

#### 3.3.7. Implications of Parabens in Pregnancy

Pharmacokinetic in vivo research has suggested placental accumulation of these substances, specifically of ethyl-paraben, when administering high doses [21]. In vitro studies on human cell lines showed that butyl-paraben can inhibit trophoblasts proliferation as well as induce their apoptosis [168]. Very few epidemiologic studies have assessed placental outcomes when it comes to paraben exposure, mainly detecting placental growth alterations and low placental weight [50]. In addition, a negative association between ethyl-paraben in cord blood and testosterone levels was found, which might be related to potential susceptibility to impaired prenatal development [30]. Recently, a study assessing the effects of different EDCs on offspring birth size outcomes found out that medium-to-high exposure to methyl-paraben was associated with lower birth weight and length in males, even though no significant association was reported for female newborns [72]. Additionally, another study pointed out that maternal urinary paraben levels were slightly inversely related to gestational length and head newborn circumference, being methyl-paraben and propyl-paraben inversely associated with body length and birth weight just in females [73]. As for GDM, even though no significant association has been found in overall population, higher levels of this propyl-paraben were detected among the overweight/obese pregnant women, a subgroup characterized for being more prone to developing GDM [75].

### 3.4. EDCs and Advanced Maternal Age

The role that advanced maternal age (AMA) may play in pregnancy has been studied to some extent, being associated with adverse fetal growth outcomes such as congenital abnormalities [169], growth restriction [170], preterm delivery [171], and maternal complications, including preeclampsia [172], GDM [173], and increased risk of caesarean delivery [174]. AMA is considered when the mother is ≥35 years old at the time of delivery [175], while very advanced maternal age (VAMA) and extremely advanced maternal age (EAMA) are established at ≥40 years and ≥45 years old, respectively [176]. These situations are becoming increasingly frequent in the last decades since pregnancies are being postponed in exchange of prioritizing education/career and owing to economic issues and poor work conditions [177]. Some studies suggest that AMA negative impact in pregnancy may be mixed, and influenced by pre-existing medical conditions, obstetric history, and maternal social characteristics (not associated with age), which arise the question of whether AMA is an independent determinant of birth outcomes [178]. As stated before, EDCs’ relevancy in pregnancy physiology, outcomes and postnatal diseases has been deeply studied, and the obtained results seem to be in line with the ones extracted by AMA research. However, the relationship between EDCs and AMA has been scarcely addressed, being mainly focused on fertility. For instance, a recent study has assessed the relationship between several EDCs and fertility in two groups couples: <35 years old and ≥35 years old. Overall, age did not seem to modify the association between BPA, phthalates, and benzophenone (BP) type UV filters. Nevertheless, benzophenone 2 (BP-2) and 4-hydroxybenzophenone (4OH-BP) reported longer time-to-pregnancy (TTP) among females ≥ 35 years old, which reflected 39% and 29% decreases in fertility, respectively for each chemical [74]. Another study found out that both AMA and parental occupational exposure to EDCs were associated with increased risk of cryptorchidism in the offspring [76]. Another study concluded that elevated levels of triclosan and some phthalates may be associated with diminished fecundity and shorter time to pregnancy, which was also correlated with AMA. However, they failed to find this association regarding BPA [179].

Lipophilic structures are rather frequent in several types of EDCs, which makes them easily accumulable in the adipose tissue, especially for those catalogued as persistent organic pollutants. Within this context, EDCs would have the potential for a “vicious spiral” related to their obesogenic effect, as they would increase fat storage, which would be followed by greater retention of lipophilic EDCs. Continuous exposure over the years would increase their own accumulation, which in turn, would translate to higher levels of these compounds in older subjects (pregnant women in this case) [180]. On the other hand, age is frequently associated with weight gain and changes in body composition characterized by increased fat mass, which would make it easier to accumulate increased levels of EDCs, potentially dangerous for the gestation process. Additionally, women’s bodies are characterized by a higher fat content compared to men, and pregnancy is a physiological state also related to important weight and fat composition changes [181]. On the other hand, age affects the metabolism of several EDCs, especially PAEs, which would consequently lead to greater distribution, accumulation, and eventually, affectation by these substances, as the aged body loses some of its capacity to eliminate them [182]. These combined factors would make of AMA women particularly prone to EDCs accumulation, potentially worsening not only the course of the gestation process, but also augmenting the probability of detrimental consequences related to “early programming” effects and appearance of NCDs in postnatal life [8].

Despite the lack of research that considers both the study of AMA and EDCs during pregnancy, it seems reasonable to expect worse outcomes when both factors are present during the gestation process, even though it remains unclear whether the negative impact of AMA may be partially explained by greater EDCs concentrations intrinsically associated to older mothers.

### 3.5. EDCs Exposure Prevention

According to all of the information addressed in previous sections, and even though there is a need to gather more data regarding EDCs and pregnancy, reducing the exposure to these molecules would be a fundamental strategy to prevent their possible detrimental effects in both mothers and their offspring. The main EDCs source in daily life undoubtedly comes from the diet, including food itself, processing practices, packaging, and cooking methods. In the case of phthalates, their concentrations may vary due to several aspects, especially food lipid content. It seems that, owing to their lipophilic features, animal-derived food constitutes a major source of phthalates, especially those with higher fat content, as these EDCs can easily bioaccumulate in them [183]. As for other chemicals, consumption of frozen or canned seafood has demonstrated to be one of the main sources of both BPA and PFCs during pregnancy [4]. On the contrary, eggs, milk, and yogurt contain low levels of EDCs, especially phthalates. Regarding fruits and vegetables, they are associated with low exposures (except for soy, its derivatives, and other vegetables rich in phytoestrogens), as well as pasta and rice, if they are not presented in cans and/or jars [184].

Regarding meal preparation, it is also relevant to consider the type of cookware, as some of their coatings have PFOA in their composition, especially if it is old and has not been recently renewed. In addition, food contact materials are an extensive source of EDCs such as DEHP, such as blisters, films, bottles, trays, screws, and transport packaging, even though their use has drastically dropped in Europe in the last years. On the other hand, BPA is necessary for the manufacture of food contact materials and epoxy resins (which are applied on cans and other recipients) made of polycarbonate plastic. This constitutes the major source of dietary BPA exposure, as residual monomers may migrate from these plastics to foods and beverages. Another issue related to EDCs generation and exposure are cooking methods. PAHs are formed during high temperature combustion processes, so they are more likely to appear during grilling, barbecuing, or smoking [4]. Taking all these facts into consideration, several practical arrangements can be made to reduce EDCs exposure during reproductive age and pregnancy, to diminish the risk of negative outcomes that may compromise the success of the gestation process for both the mother and the offspring.

When it comes to food selection, there are some choices that should be preferably made. On the one hand, seasonal food would be mostly advisable, especially for fruits, vegetables, and fish. Consumption of canned/frozen fish and seafood should be reduced to once a week. Glass jars are the best choice regarding legumes and sauces, rather than cans. On the other hand, tap water is preferable, and when using beverage containers, glass bottles should be chosen over those made of plastic. Ready-made food should be avoided, especially if it requires heating, as well as boxes, wrappers, and popcorn bags. As for coffee and tea, alternatives to plastic coffee makers and tea bags must be taken into consideration. It is also relevant to turn the attention to products’ precedence, especially when they come from non-European countries without CE mark [185,186].

With regard to the cooking process, some precautions should be made. Firstly, barbecuing and grilling, especially over charcoal, must be avoided, prioritizing other cooking methods, and ensuring proper kitchen ventilation. Removing meat fatty portions before cooking would be also advisable. Secondly, burned, and charred food parts must be removed, and smoked foods should be limited to once a month. Finally, worn non-stick cookware must be discarded, making use of undamaged containers, especially if they are aimed to heat food. These must be used following the specifications of the manufacturer, avoiding polycarbonate plastics within the microwave. Instead of them, porcelain, glass, and stainless-steel containers should be prioritized [4,185].

Storage and container care must also be considered. Dishwashers must be used just for those plastics suitable for high temperatures. In addition, food should not be stored in damaged plastic containers. It is also important to let hot food cool before pouring it in plastic containers not suitable for heat. As for material choices, PVC containing DEHP must be limited, also preferring PAEs- and BPA-free products [4,186].

Making bigger efforts to forewarn mothers of these recommendations seems to be of particular relevance, as recent surveys carried out in France and Canada point out that more than half the women interviewed during pregnancy or after delivery, had never heard of endocrine disruptors [187,188]. This scarce knowledge of EDCs risks has been related to the limited and even non existing time that doctors who follow mothers during gestation dedicate to instruct them on environmental risks’ prevention [189]. Therefore, it appears logical to claim that legislation and public health programs adopted by governments should be accompanied by awareness campaigns and proper training for doctors, especially obstetricians, in this field.

## 4. Limitations of the Study

The study of endocrine disruptors is a difficult subject, which gets complicated if their effects are intended to be determined in physiologic situations such as pregnancy. There are several limitations that may explain the contrasting results obtained by the previously addressed studies. Firstly, there is a high variability of experimental models that can be applied, except from clinical trials since the interventions required are not ethical to be performed in humans. Secondly, exposure can be evaluated in a wide range of conditions, considering concentration, timing, and distribution, as well as the inter-relationships when it comes to the effects of mixtures. Many articles limit their examination to a single class of chemical or its metabolites, due to the complexity to manage the actual exposure existing in the human body, the cost of analytical technologies, and the large sample size required to sufficiently power interaction testing, which has restricted the needed simultaneous evaluation of the thousands of natural and synthetic compounds with endocrine effects. Another limitation to highlight is the existence of some imprecision in the assessment of those EDCs with a shorter half-life. For example, for non-persistent substances such as BPA with such variable levels, studies relying on a certain spot or period during pregnancy are likely to have lower power and strong attenuation bias. In this sense, it would be advisable to collect frequent samples across the gestation process to reduce measurement errors. Another issue is the impossibility to measure EDCs directly in target tissues when it comes to human research, or at least, not during the pregnancy process (placentas can only be assessed after delivery). On the other hand, the use of supraphysiological dosing regimens in animal research reports direct cytotoxic outcomes. However, magnitudes are frequently higher than human exposures, so their biomonitoring and pharmacokinetic information should be used to establish predictive physiologically based models that allow the use of comparable doses in animals. The current gaps in knowledge about metabolism and distribution of some chemicals during gestation is another limitation to take into consideration. Finally, the different level of research among EDCs makes it difficult to draw conclusions regarding the less addressed chemicals compared to those widely evaluated, such as BPA or some phthalates.

## 5. Conclusions

The numerous ways for the population to get directly or indirectly in contact with EDCs, together with the demonstrated negative impact and toxicity of these substances on reproductive health and pregnancy development, highlights the need to reevaluate their use in the current society. During the last decades, a large amount of epidemiological and animal-based studies has pointed out an association between EDCs exposure and these events, sometimes in a sex-specific way. Bisphenols are the group of compounds with a higher level of evidence regarding pregnancy alterations and outcomes, being related with several structural and functional defects in the placenta, possibly leading to a greater risk of complications such as preeclampsia and pregnancy loss (the association with others such as GDM or intrauterine growth restriction and preterm delivery is not consistent in these studies). Phthalates have also been deeply studied due to their implications in the appearance of abnormalities during gestation, specially related to PPAR-γ activation and thyroid disruption. For other EDCs such as pesticides, PAHs, and PFCs, even though the scientific evidence available is limited, have also been related with detrimental effects in pregnancy. As for phytoestrogens and parabens, there is a gap of knowledge on their association with placental alterations and gestational outcomes. It cannot be ignored that some of the studies included in this review yield heterogeneous results, some of them contradictory, or do not show statistical significance in the association between some chemicals and pregnancy outcomes. According to this and bearing in mind the complexity of these studies to demonstrate a precise cause-effect relationship, further research needs to be carried out to clarify the actual effects of EDCs on gestation. A better understanding of the unknown mechanisms of action involved still constitute a challenge for the scientist community, for which new approaches are needed. On the other hand, some replacements have emerged as a solution to mitigate the demonstrated effects of their congeners. Despite the limited available research on these novel molecules, several studies highlight detrimental repercussions similar or even worse to those of the EDC they try to replace, thus pointing out this may not be an efficient solution. For all these reasons, the impact of EDCs on pregnancy must raise concerns about the adequate use of common daily products, encouraging preventative measures to avoid their detrimental implications.

## Figures and Tables

**Table 1:** Summary of the most relevant EDCs regarding pregnancy development and their exposure sources.

EDC Group	Molecules	Exposure Sources
Bisphenols	Bisphenol A (BPA), Bisphenol S (BPS), Bisphenol F (BPF), Bisphenol B (BPB), Bisphenol AF (BPAF), Tetramethyl bisphenol F (TMBPF), Tetrabromo bisphenol A (TBBPA).	Food packaging, bottles, coat metal products, cans, dinnerware, coating powders, medical material, dental sealants, thermal printing papers.
Phthalates (PAEs)	Primary molecules: Diethyl phthalate (DEP), Di(2-ethylhexyl) phthalate (DEHP), Di(2-propylheptyl) phthalate (DPHP), Di-iso-nonyl phthalate (DINP), Benzyl butyl phthalate (BBP), Di-n-butyl phthalate (DBP), Dimethyl phthalate (DMP), Di-n-octyl phthalate (DNOP), Diisononyl cyclohexane dicarboxylate (DINCH). Metabolites: Monoethyl phthalate (MEP), Mono(2-ethyl-5-hydroxyhexyl) phthalate (MEHHP), Mono(2-ethylhexyl) phthalate (MEHP), Mono(2-ethyl-5-oxohexyl) phthalate (MEOH), Mono(2-ethyl-5-carboxypentyl) phthalate (MECPP), Mono(3-carboxypropyl) phthalate (MCPP), Mono-iso-butyl phthalate (MIBP), Monobutyl phthalate (MBP), Mono-n-butylphthalate (MNBP), Mono-benzyl phthalate (MBZP), Mono-(2-ethyl-5-oxohexyl) phthalate (MEOHP), Monocarboxy-isononly phthalate (MCNP), Mono-carboxy-isooctyl phthalate (MCOP).	Food packaging, pharmaceutical coatings, personal care products (perfumes, deodorants, soaps, shampoos, lotions), toys, textiles, building materials, medical equipment.
Pesticides	Organochlorine pesticides (OCPs): DDT, hexachlorobenzene, dieldrin, lindane. Organophosphates (OPs): Parathion, Methyl Parathion, Malathion. Pyrethroids: Fenvalerate, Permethrin, Deltamethrin.	Insects, fungus, and weeds control.
Polycyclic aromatic hydrocarbons (PAHs)	Polychlorinated biphenyls (PCBs) Polybrominated diphenyl ethers (PBDEs)	Building materials, electrical equipment, paints, textiles, furniture, foams, hydraulic fluids, combustion processes.
Perfluorinated alkylated substances (PFASs)/Perfluorinated compounds (PFCs)	Perfluorooctane sulfonate (PFOS), Perfluorooctonoic acid (PFOA), Perfluononanoic acid (PFNA)	Non-stick cookware, firefighting foams, waterproof clothing, personal care products, anti-fouling paints.
Parabens	Methyl-paraben, Ethyl-paraben, Propyl-paraben, Butyl-paraben	Personal care products.
Phytoestrogens	Isoflavones: Genistein, Daidzein, Glycitein. Coumestans: Coumestrol. Lignan	Natural: soy (and derivate products), beans, other legumes, grains, fruits, vegetables.

**Table 2:** Summary of major findings in articles included and reviewed.

Reference	EDC	Study Design	Major Findings
Rodríguez-López et al., 2021 [33]	PAEs (DNBP, DEHP), BPA, butylparaben	Animal model	Exposure to an EDC mixture during pregnancy exhibits multi- and transgenerational disruption of sexual maturation (folliculogenesis) and maternal behavior through a hypothalamic epigenetic reprogramming.
Liu et al., 2021 [34]	BPA	Animal model	Low BPA doses can reduce mice sperm quality by altering germ cell proliferation, leading to decreased fertility.
Mantzouki et al., 2019 [35]	BPA	Observational	High concentrations of BPA could contribute to male infertility (conflicting results).
Song et al., 2020 [36]	BPA	Animal model	BPA treatment reduces placental efficiency and fetal weight, increasing inflammatory and oxidative stress biomarkers, through epigenetic changes.
Ye et al., 2019 [37]	BPA	Animal model	BPA exposure disrupts trophoblast cell invasion and produces abnormal placental vessel remodeling which leads to preeclampsia-like characteristics.
Kaimal et al., 2021 [38]	Bisphenols (BPA, BPS, BPF)	Animal model	Prenatal exposure to BPF affects pregnancy outcomes (increasing spontaneous abortions), BPS alters male anogenital distance, and all three bisphenols alter ovarian function in female offspring.
Ticiani et al., 2021 [39]	BPS	In vitro	BPS exposure at environmentally relevant levels might result in placenta dysfunction, affecting fetal development, through a blockage of epidermal growth factor (EGF) binding.
Harnett et al., 2021 [40]	Bisphenols (TMBPF, BPA, BPS, BPF)	Animal model	TMBPF is the second-most toxic and teratogenic of the molecules tested (BPAF > TMBPF > BPS > BPA). BPA replacements exert adverse effects on early embryo development, having implications for reproductive health.
Narciso et al., 2019 [41]	BPA	In vitro	Placenta is a target organ of BPA, modifying the expression of hormones and proteins related to trophoblast fusion and apoptosis. This could bring possible implications on fetal and pregnancy health.
Amin et al., 2021 [42]	BPA	Observational	There is no significant association between BPA levels and anthropometric measurements, nor gestational age. No significant relationship exists between BPA and β-hCG with birth outcomes. This lack of association may be due to the low levels of urinary BPA (adverse effects might be related to higher concentrations) (conflicting results).
Huang et al., 2019 [43]	BPA, BPS	Observational	Higher average concentrations of BPA across pregnancy are related to a 1.97-day decrease in the gestation process. BPA levels in three trimesters are also negatively correlated with gestational age and positively associated with preterm birth.
Namat et al., 2021 [44]	BPA	Meta-analysis	Higher BPA exposure is related to increased risk of preterm birth and reduced gestational age, suggesting that exposure in the third trimester may be a critical period.
Mustieles et al., 2019 [45]	PAEs (DEHP, MEP)	Observational	Certain paternal and maternal urinary phthalates might affect placental weight and birth weight/placental weight ratio.
Kim et al., 2021 [46]	Bisphenols (BPA, BPS, BPF)	Observational	There seems to be an inverse association between bisphenol mixtures and birth weight.
Philips et al., 2020 [47]	Bisphenols, PAEs	Observational	High maternal urine concentrations of bisphenols in early pregnancy leads to reduced gestational weight in the second half of gestation. No association was found for PAEs.
Tang et al., 2023 [48]	Bisphenols (BPA, BPS, BPF, BPB, TBBPA)	Observational	BPS shows a positive effect on the risk of gestational diabetes mellitus, whereas BPA and TBBPA have a negative effect on it. Exposure to the mixture of the five bisphenols was negatively related to the risk of gestational diabetes (conflicting results)
Derakhshan et al., 2021 [49]	PAEs (DEHP, DINP, DBP, DINCH)	Observational	Exposure to phthalates may interfere with the thyroid system during gestation, even for those compounds introduced to replace known disruptive phthalates.
Philippat et al., 2019 [50]	PAEs (MCNP, MCOP), BPA, parabens	Observational	There is a positive association between the sum parabens and placental weight, also providing preliminary evidence of possible relationship between MCNP, MCOP and both placental weight and placental–to–birth weight ratio.
Shoaito et al., 2019 [51]	PAEs (MEHP)	In vitro	MEHP produces significantly lower PPAR-γ activity and less villous cytotrophoblast differentiation, whereas high doses have the opposite effect. MEHP also inhibits hCG production and has significant effects on the mitogen-activated protein kinase (MAPK) pathway.
Chang et al., 2021 [52]	PAEs (DEHP)	Observational	Women patients with recurrent pregnancy loss (RPL) have a significantly higher cumulative exposure to phthalates than controls. The risk of RPL is strongly associated with the higher quartiles of DEHP.
Guo et al., 2022 [53]	PAEs (BBP, DEHP, DMP, MBP)	Observational	Exposure to BBP, DEHP, and DMP are significantly positively associated with the risk of fetal growth restriction, while MBP showed a negative relationship, only among girls (conflicting results).
Welch et al., 2022 [54]	PAEs	Observational	Increases in urinary levels of phthalate metabolites show an association with higher odds of preterm birth (from 12% to 16%).
Zhang et al., 2020 [55]	PAEs (DEHP)	Observational	Maternal higher exposure to DEHP metabolites augment the risk of preterm delivery.
Philips et al., 2019 [56]	Bisphenols, PAEs	Observational	Increases in high molecular weight phthalate metabolites are associated with higher early pregnancy sFlt-1/PlGF ratio (related to preeclampsia). BPA is associated with higher intercept and reduced slope of the umbilical and uterine artery PI Z-score.
Bedell et al., 2021 [57]	PAEs (MEP, MCPP, MIBP, MBP, DEHP)	Observational	Higher levels of first trimester MEP and MCPP, and third trimester MIBP, are significantly related to diagnosis of pregnancy-induced hypertension. First-trimester MBP and MEP, along with DEHP, are each associated with augmented systolic blood pressure across pregnancy.
Hirke et al., 2023 [58]	PAEs (MEP) PFAS	Meta-analysis	Among several PAEs analyzed, MEP is the only one that showed a positive relationship in this regard with gestational hypertension.
Shaffer et al., 2019 [59]	PAEs	Observational	There is an association between MEP and gestational diabetes mellitus. Other phthalate metabolites are linked to impaired glucose intolerance, with possible stronger relationships in certain racial/ethnic groups.
Yan et al., 2022 [60]	PCBs, BBDEs, PAEs, PFAS	Meta-analysis	Exposure to certain PCBs, PBDEs, PAEs, and PFAS increase the risk of gestational diabetes.
Liang et al., 2022 [61]	PAEs (MEHP)	Observational	Patients with gestational diabetes have higher MEHP levels than those in the control group. The diabetes and MEHP dose-response associations are different among pregnant women aged < 35 years and those aged > 35 years.
Zukin et al., 2021 [62]	PAEs	Observational	There is no association between prenatal phthalate levels and increased risk of hyperglycemia, impaired glucose tolerance, or gestational diabetes mellitus. However, there is an increased odds of excessive gestational weight gain, a gestational diabetes risk factor (conflicting results).
Anand et al., 2019 [63]	OCPs (DDT, lindane)	Observational	Mean levels of pesticides (DDT and lindane) are higher in the placenta of the women with preterm birth. Exposed women are more likely to deliver a preterm baby than not exposed ones. Increasing maternal age reduces the risk of preterm delivery.
Pearce et al., 2021 [64]	OCPs, PBDEs, PCBs, PFAS	Observational	EDCs combinations with high PBDEs levels are related to low birth weight, and combinations with high concentrations of PCBs and PFAS are associated with augmented birth weight.
Robinson et al., 2019 [65]	PBDEs	In vitro	PBDEs significantly reduce primary villous cytotrophoblasts viability and increased death, also decreasing their ability to migrate and invade, which could adversely impact placental activity.
Wang et al., 2022 [66]	PBDEs	Observational	PBDEs are related to shorter gestation and higher risk of certain preterm birth subtypes among no-obese pregnant women.
Kelley et al., 2019 [67]	PAEs	Observational	Maternal and cord blood cytokines are differentially associated with individual and mixtures of EDCs, being several of them related to gestational age and birth weight.
Liu et al., 2019 [68]	PFAS	Observational	There seems to be a structure-specific association between short-chain PFAS exposure and both gestational diabetes risk and impaired glucose homeostasis in pregnant women.
Rahman et al., 2019 [69]	OCPs, PBDEs, PCBs, PFAS	Observational	Environmentally relevant concentrations of PCBs and some PBDEs and PFAS are associated with gestational diabetes.
Huang et al., 2019 [70]	PFAS	Observational	Prenatal PFAS exposure seems to be positively associated with the risk of preeclampsia and overall hypertensive disorders of pregnancy.
Awobajo et al., 2022 [71]	Genistein	Animal model	Serum IGF-1 and PIGF are increased when genistein is administered at gestational day 12 and 16. However, serum IGF-1 and PIGF levels are reduced in the placenta at gestational day 20. Placental sFLT-1 levels are increased gestational day 12 and 20. The sFL-1/PlGF ratio in exposed placenta samples decreases at gestational day 16 increases at gestational day 20, whereas the opposite is reported in serum (conflicting results)
Uldbjerg et al., 2022 [72]	Parabens, PAEs (MEP), BPA	Observational	BPA exposure is associated with lower birth size in male offspring. Similarly, exposure to MEP in male offspring is also related to lower birth weight. No associations are found regarding parabens nor female offspring.
Pacyga et al., 2022 [73]	Parabens	Observational	Maternal urinary paraben concentrations are modestly inversely associated with newborn head circumference and gestational length. Methylparaben and propylparaben seem to be inversely related to birth weight, body length, and weight/length ratio just in female offspring. Maternal diet may modify associations between parabens and birth size in a sex-specific way.
Pollack et al., 2022 [74]	BPA, PAEs, Benzophenones	Observational	Age does not seem to modify the association between BPA, phthalates, and benzophenone (BP) type UV filters. However, benzophenone 2 (BP-2) and 4-hydroxybenzophenone (4OH-BP) report longer time-to-pregnancy among females ≥ 35 years old, which reflected 39% and 29% decreases in fertility, respectively for each chemical.
Li et al., 2019 [75]	Parabens	Observational	Even though no significant association is found in overall population, higher levels of this propyl-paraben are detected among the overweight/obese pregnant women (who are more prone to developing GDM).
Estors Sastre et al., 2019 [76]	BPA, PAEs	Observational	Both AMA and parental occupational exposure to EDCs are associated with increased risk of cryptorchidism in the offspring.

## Data Availability

Not applicable.
